# The Topological Analysis of the ELF_x_ Localization Function: Quantitative Prediction of Hydrogen Bonds in the Guanine–Cytosine Pair

**DOI:** 10.3390/molecules26113336

**Published:** 2021-06-01

**Authors:** Johanna Klein, Paul Fleurat-Lessard, Julien Pilmé

**Affiliations:** 1 Sorbonne Université, Laboratoire de Chimie Théorique, UMR 7616 CNRS, CC 137, 4, Place Jussieu F, CEDEX 05, 75252 Paris, France; johanna.klein@sorbonne-universite.fr; 2 Institut de Chimie Moléculaire de l’Université de Bourgogne (ICMUB), CNRS UMR 6302, 9 Avenue Alain Savary, BP 47870, CEDEX, 21078 Dijon, France; Paul.Fleurat-Lessard@u-bourgogne.fr

**Keywords:** ELF, ELF_x_, DNA, hydrogen bond, nucleophilic, electrophilic, base pair, cytosine, guanine

## Abstract

In this contribution, we recall and test a new methodology designed to identify the favorable reaction pathway between two reactants. Applied to the formation of the DNA guanine (G) –cytosine (C) pair, we successfully predict the best orientation between the base pairs held together by hydrogen bonds and leading to the formation of the typical Watson Crick structure of the GC pair. Beyond the global minimum, some local stationary points of the targeted pair are also clearly identified.

## 1. Introduction

Among numerous ideas published by Linus Pauling, he proposed with Robert B. Corey in 1953 a pioneering triple DNA helix structure with the bases on the outside [1]. 

**Figure 1:**
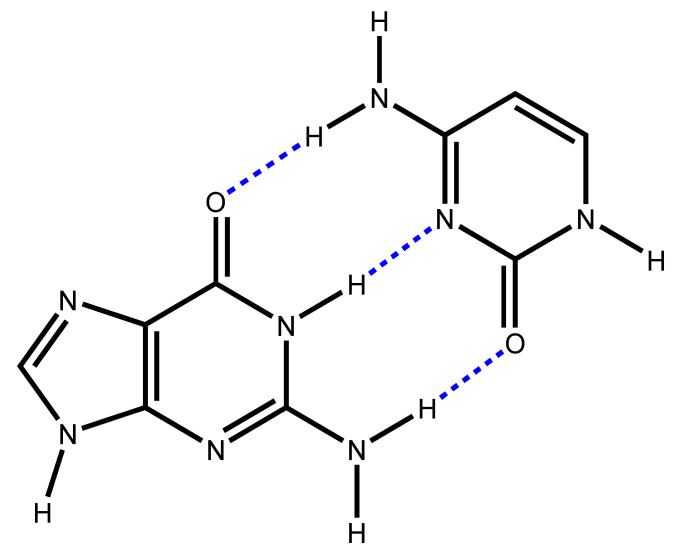
Guanine (G) cytosine (C) pair structure. The intermolecular hydrogen-bonds are displayed in blue.

Although this Pauling’s structure soon turned out to be false, this work has paved the way for the discovery of DNA’s double-helix structure [2]. In recent decades, DFT quantum chemical studies of the Watson Crick base pairs investigated the geometry, the energy and other typical properties of the hydrogen bonds (HB) that hold together adenine–thymine (AT) and guanine–cytosine (GC) pairs [3,4,5,6,7,8]. Note that L. Pauling and R. B. Corey have already highlighted the role of hydrogen bonding in proteins [9]. The main concern assessed in this work is related to the validity of the molecular orbital point of view regarding the geometries of these base pair systems where hydrogen bonds play a crucial role. Indeed, it has been shown that the stability of the Watson Crick base pairs is related to a charge-transfer due to donor/acceptor orbital interactions (oxygen and nitrogen lone pairs, N-H σ* character) [10]. For example, consider the well-known most stable pair structure guanine (G)–cytosine (C), as depicted in Figure 1 [7,10,11,12].

In this article, we tackle the possibility to find the guanine (G)–cytosine (C) pair geometry simply by looking at the orientation of the donor and acceptor domains of the bases.

## 2. Electron Localization Function for Chemical Reactivity

Nowadays, the topological analysis of the electron localization function (ELF) is a well-established tool to describe both covalent and non-covalent interactions [13,14,15,16,17,18,19,20]. However, the tricky question is to determine the most favorable relative orientations between the ELF topological domains of two reactants and, thus, to identify the preferred pathways when both molecules approach each other remains a tremendous challenge. Intuitively, it is established that favorable chemical reactions happen when electron acceptor and electron donor domains are adequately oriented. Recently, we have proposed a methodology designed to identify the favorable orientations between two reactants [21]. In this work, the topological domains are the ones of the modified ELF, termed ELF_x_ [22] defined from ELF as follows:

(1) $$\chi_{x}(r)=\frac{\chi (r)}{2\;x(r)}{\;\mathrm{and}\;\mathrm{ELF}}_{x}(r)=\frac{1}{{1+\chi }_{x}{\left(r\right)}^{2}}$$

The kernel χ(r) of ELF being defined as:

(2) $$\chi (r)\;=\;\frac{\tau_{N}(r)\;-\;\frac{1}{8}\frac{{\left|\nabla \rho {\left(r\right)}_{N}\left(r\right)\right|}^{2}}{{\rho \left(r\right)}_{N}}}{c_{F}\rho {\left(r\right)}_{N}{}^{5/3}}\;$$

where $c_{F}=\frac{3}{10}{\left(3\pi^{2}\right)}^{\frac{2}{3}}$ is the Fermi constant, $\tau_{N}\left(r\right)$ is the positive definite kinetic energy density and $\rho {\left(r\right)}_{N}$ is the total electron density of a molecular system with N electrons. x(**r**) is a normalized dimensionless quantity that can be expressed from the field of the frontier molecular orbitals [23].

(3) $$x(r)=\frac{\rho {\left(r\right)}_{\mathrm{HOMO}}}{\rho {\left(r\right)}_{N}}\;\mathrm{or}\;x(r)=\frac{\rho {\left(r\right)}_{\mathrm{LUMO}}}{\rho {\left(r\right)}_{N+1}}$$

$\rho {\left(r\right)}_{N+1}$ is the total electron density of the molecular system with N + 1 electrons with the same geometry and the same orbitals that are obtained for the system with N electrons. In this latter case where $x(r)=\frac{\rho {\left(r\right)}_{\mathrm{LUMO}}}{\rho {\left(r\right)}_{N+1}}$, $\rho {\left(r\right)}_{N+1}$ (and $\tau_{N+1}\left(r\right)$) are also consistently used for the calculation of the ELF kernel.

**Figure 2:**
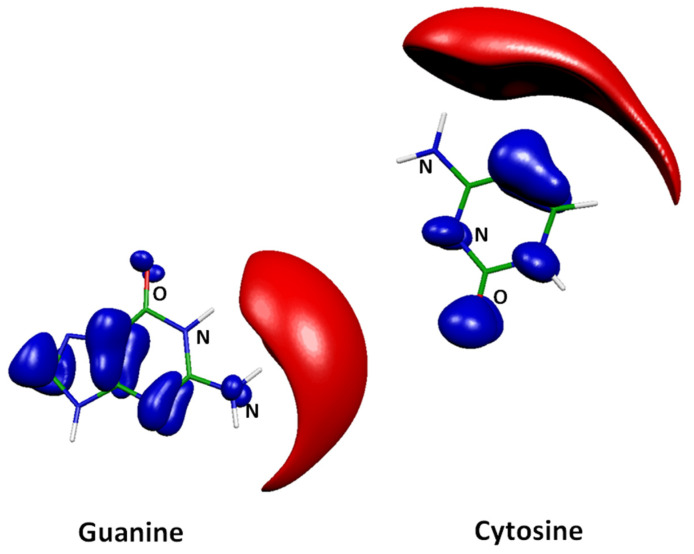
Main ELF_x_ localization domains of the guanine and cytosine molecules computed at the M06-2X/6-311++G(3df, 2pd) level of theory. Color Code: blue: nucleophilic regions and red: electrophilic regions. Carbon atoms are in green, Nitrogen atoms in blue, Oxygen atoms in red and Hydrogen atoms in white.

The ELF_x_ localization domains are well suited for describing the chemical reactivity between donors and acceptors because they match with the electrophilic and the nucleophilic regions which are spread out over the molecular space. This is illustrated in Figure 2, which represents the ELF_x_ domains of guanine and cytosine.

The ELF_x_ topological analysis of the guanine and cytosine molecules obtained in their isolated states yields, respectively, to valence basins accounting for electrophilic basins (red domains) and several nucleophilic basins (blue domains). The outside domains around hydrogen atoms appear as electrophilic while domains around nitrogen atoms as well as the oxygen lone-pairs clearly have a nucleophilic character.

## 3. Theoretical Model

### 3.1. Coulomb Intermolecular Interaction Energy

The total energy of a molecule or a complex can be split within the framework of the interacting quantum atoms (IQA) [24,25]. The IQA coulomb contribution between two molecules MA et MB, here termed $E_{\mathrm{MA}-\mathrm{MB}}^{\mathrm{Coul}}$, reads:

(4) $$E_{\mathrm{MA}-\mathrm{MB}}^{\mathrm{Coul}}=\underset{\Omega_{A}\in (4)\mathrm{MA}}{\sum }\underset{\Omega_{B}\in \mathrm{MB}}{\sum }\left[\int_{r_{A}\in {\;\Omega }_{A}}\;\int_{r_{B}\in \Omega_{B}}\frac{\left[Z_{A}\;\delta \left(r_{A}-R_{A}\right)-\;\rho \left(r_{A}\right)\right]\;\left[Z_{B}\;\delta \left(r_{B}-R_{B}\right)-\;\rho \left(r_{B}\right)\right]}{\left|r_{A}-{\;r}_{B}\right|}{\;dr}_{A}{dr}_{B}\right]$$

$\left|r_{A}-r_{B}\right|$ being the distance between an electron in the domain Ω_A_ and an electron in the domain Ω_B_, respectively. **R**_A_ and **R**_B_ are the nuclear locations of atoms A and B belonging to $\Omega_{A}$ and $\Omega_{B}$ domains with charges Z_A_ and Z_B_. When MA and MB are located far from each other, we assume that $E_{\mathrm{MA}-\mathrm{MB}}^{\mathrm{Coul}}$ accounts for a large fraction of the total interaction energy [21].

### 3.2. Electron Transfer

The coulomb energy stabilization between an electron donor (MA) and an electron acceptor (MB) can be evaluated by the first-order variation of $E_{\mathrm{MA}-\mathrm{MB}}^{\mathrm{Coul}}$ expressed in terms of the response to changes in the number of electrons ΔN_A_ or ΔN_B_ where the external potential remains unchanged:

(5) $$\Delta E_{\mathrm{MA}-\mathrm{MB}}^{\mathrm{Coul}}={\left(\frac{\partial E_{\mathrm{MA}-\mathrm{MB}}^{\mathrm{Coul}}}{\partial N_{A}}\right)}_{N_{B}}\Delta N_{A}+{\left(\frac{\partial E_{\mathrm{MA}-\mathrm{MB}}^{\mathrm{Coul}}}{\partial N_{B}}\right)}_{N_{A}}\Delta N_{B}{=E}_{\mathrm{dual}}^{\mathrm{MA}/\mathrm{MB}}\;\Delta N_{A}$$

The total variation $\Delta N={\Delta N}_{A}+{\Delta N}_{B}=0$ because the total system is isolated. ${\;E}_{\mathrm{dual}}^{\mathrm{MA}/\mathrm{MB}}$ is negative when the electron transfer goes spontaneously from MA (nucleophile) to MB (electrophile). After some developments previously detailed elsewhere [21], we obtain:

(6) $${\;E}_{\mathrm{dual}}^{\mathrm{MA}/\mathrm{MB}}=\underset{\Omega_{A}\in \mathrm{MA}}{\sum }\underset{\Omega_{B}\in \mathrm{MB}}{\sum }\left[\int_{r_{A}\in {\;\Omega }_{A}}\int_{r_{B}\in \Omega_{B}}\frac{{\;f\;(r}_{B})\;\left[{\;Z}_{A}\delta \left(r_{A}-R_{A}\right)-{\rho (r}_{A})\right]-{\;f\;(r}_{A})\;\left[{\;Z}_{B}\delta \left(r_{B}-R_{B}\right)-{\rho (r}_{B})\right]}{\left|r_{A}-r_{B}\right|}{dr}_{A}{\;dr}_{B}\;\right]$$

where ${f(r}_{A})$ and ${f(r}_{B})$ are the Fukui functions [23] typically associated with reactive nucleophilic or electrophilic sites of the reactants.

The choice of the condensation scheme remains arbitrary as far as that of an electron domain or the definition of an atom in a molecule remains arbitrary. Here, we can clearly dissociate the MA and MB domains where electrophilic and the nucleophilic regions are spread out over their respective molecular space. This typically matches with the topological partition of the electron localization function ELF_x_.

### 3.3. Practical Interactions Model

Equation (4) is exact but can be computationally expensive. In practice, it can be numerically evaluated by means of a multipole expansion (ME) [26]. We use only the first terms of the ME (that is only the monopoles). For the general case in which both molecules MA and MB exhibit some donor and acceptor sites, the monopoles’ development leads to the compact equation:

(7) $${\;E}_{\mathrm{dual}}{\;=\;E}_{\mathrm{dual}}^{\mathrm{MA}/\mathrm{MB}}{+\;E}_{\mathrm{dual}}^{\mathrm{MB}/\mathrm{MA}}=\;\overset{}{\underset{\Omega_{A}\in \mathrm{MA}}{\sum }}\overset{}{\underset{\Omega_{B}\in \mathrm{MB}}{\sum }}[\frac{{\;2\;N}_{\Omega_{A,\mathrm{Nu}\;}}N_{\Omega_{B,\mathrm{Nu}\;}}}{\left|r_{\Omega_{A}}-r_{\Omega_{B}}\right|}-\frac{{\;N}_{\Omega_{A,\mathrm{Nu}\;}}N_{\Omega_{B,\mathrm{El}\;}}}{\left|r_{\Omega_{A}}-{\;r}_{\Omega_{B}}\right|}-\frac{{\;N}_{\Omega_{A,\mathrm{El}\;}}N_{\Omega_{B,\mathrm{Nu}\;}}}{\left|r_{\Omega_{A}}-r_{\Omega_{B}}\right|}+\frac{Z_{A}\left(N_{\Omega_{B,\mathrm{El}\;}}-N_{\Omega_{B,\mathrm{Nu}}}\right)}{\left|r_{\Omega_{B}}-R_{A}\right|}+\frac{Z_{B}\left(N_{\Omega_{A,El\;}}-N_{\Omega_{A,\mathrm{Nu}}}\right)}{\left|r_{\Omega_{A}}-{\;R}_{B}\right|}]$$

in which N_ΩNu/El_ are the populations of nucleophile/electrophile domains, respectively, obtained from the usual condensation of the HOMO/LUMO density computed over the ELF_x_ basin volumes [27]. $r_{\Omega_{A}}$ and $r_{\Omega_{B}}$ are the locations of basin attractors belonging to $\Omega_{A}$ and $\Omega_{B}$ domains, respectively.

## 4. Results and Discussion

**Figure 3:**
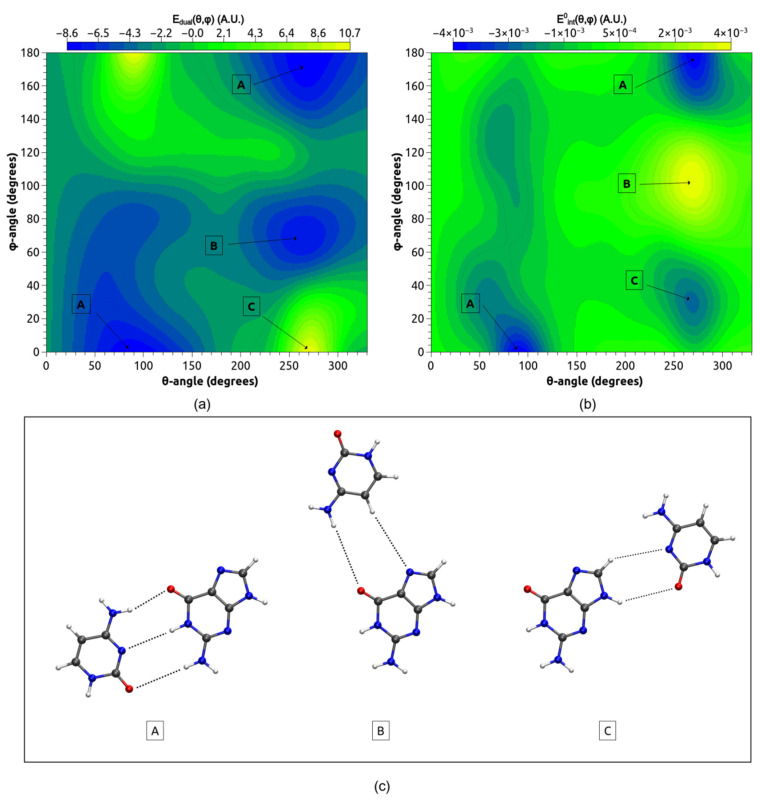
Two-dimensional map calculated at the M06-2X/6-311++G(3df,2pd) level of theory E_dual_(θ, φ) (**a**) the DFT intermolecular interaction energy $E_{int}^{0}\left(\theta ,\varphi \right)$ between the cytosine and the guanine vs. (**b**) E_dual_(θ, φ) surfaces for base pair GC formation. (**c**) Stationary points obtained from the maps, the structure (A) is the global minimum. Carbon atoms are in grey, Nitrogen atoms in blue, Oxygen atoms in red and Hydrogen atoms in white.

We explored the conformational space of the base pair GC_WC_ formation using the Equation (7) with the algorithm previously outlined elsewhere [21]. All relative rotation angles (*θ*, *φ*) of the center of mass of C around the center of mass of G have been tested, with the distance between the centers of mass of C and G being frozen to 8 Å. Note that the corresponding optimized distance between the centers of mass was found close to 6 Å at the M06-2X/6-311++G(3df, 2pd) level of theory. For a given (*θ*, *φ*) couple, the process selects the best orientation of C associated to the lowest value of E_dual_. Figure 3 displays the obtained map E_dual_(θ,φ) together with the corresponding map of the DFT intermolecular interaction energy $E_{int}^{0}\left(\theta ,\varphi \right)$ computed from the relevant isolated cytosine and guanine.

It is worth noting the good mapping of E_dual_ and the DFT intermolecular interaction energy $E_{int}^{0}\left(\theta ,\varphi \right)$. Indeed, the locations of critical points of E_dual_, notably the location of the global minimum (termed (A) on Figure 3), are in agreement with the DFT intermolecular interaction energy surface. We noted that the structure associated to the global minimum corresponds to the well-known orientation between C and G leading to the natural Watson Crick base pair GC_WC_ structure where three typical HNH∙∙∙O=C/NH∙∙∙N/C=O∙∙∙HNH intermolecular hydrogen bonds are observed. Two other stationary points (denoted by (B) and (C) on Figure 3) corresponding to already identified pair structures are also found on the E_dual_ map [7]. These latter structures highlight typical HNH∙∙∙O and CH∙∙∙N donor/acceptor interactions. The presence of structures (A), (B) and (C) are confirmed on the DFT interaction energy surface.

**Figure 4:**
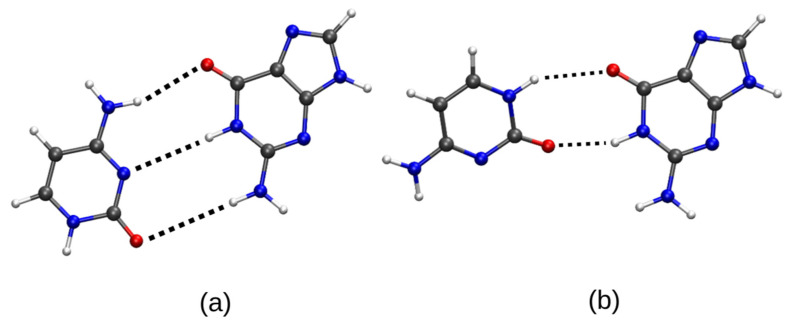
E_dua_l global minima (**a**) vs. local minima (**b**) of the guanine (G)–cytosine (C) complex. Carbon atoms are in grey, Nitrogen atoms in blue, Oxygen atoms in red and Hydrogen atoms in white.

Further analysis of the E_dual_ conformational space obtained for each given (*θ*, *φ*) couple (not only for the best orientation of C in front of G) has led us to find other local stationary points. Some of them have been previously identified in the literature [7,8]. For example, the geometry of the second lowest minimum is displayed in Figure 4b: this pair appears clearly stabilized by two symmetrically NH∙∙∙O=C hydrogen bonds.

Thus, in spite of numerous approximations used in this work, we show that the DFT energetic properties as well as the structural parameters of some identified pairs can be reasonably reproduced from our methodology.

## 5. Conclusions

The information obtained from the domains of ELF_x_ function and their populations has been used to propose an empirical model coulomb stabilization energy between electron donor and electron acceptor domains. Our methodology was able to predict the best orientations between the cytosine and the guanine leading to the formation of base pair structures. We unveil a noticeable mimicking of E_dual_ onto the DFT intermolecular interaction energy $E_{int}^{0}$. In particular, we show that the global minimum, easily identified on the E_dual_ energy surface, corresponds to the well-known Watson Crick structure for the base pair GC_WC_ in which the guanine and the cytosine molecules are held together by three hydrogen bonds (see Figure 1 and Figure 3). Some local stationary points of the GC pairs have been also identified.

## Data Availability

The data that support the findings of this study are available. Additionnal information can be requested from the corresponding author upon reasonable request.
